# Construction of a mouse model that can be used for tissue-specific EV screening and tracing *in vivo*

**DOI:** 10.3389/fcell.2022.1015841

**Published:** 2022-11-18

**Authors:** Weili Li, Jin Wang, Xiaojiao Yin, Huanhuan Shi, Benben Sun, Mengru Ji, Huichen Song, Jiachen Liu, Yihao Dou, Chenghong Xu, Xiaohong Jiang, Jing Li, Liang Li, Chen-Yu Zhang, Yujing Zhang

**Affiliations:** ^1^ State Key Laboratory of Pharmaceutical Biotechnology, Nanjing Drum Tower Hospital Center of Molecular Diagnostic and Therapy, Jiangsu Engineering Research Center for MicroRNA Biology and Biotechnology, School of Life Sciences, NJU Advanced Institute of Life Sciences (NAILS), Institute of Artificial Intelligence Biomedicine, Nanjing University, Nanjing, Jiangsu, China; ^2^ Chinese Academy of Medical Sciences, Research Unit of Extracellular RNA, Nanjing, Jiangsu, China

**Keywords:** extracellular vesicles (EVs), mouse model, CD63flag-EGFP, screening and tracing, tissue-specific EV

## Abstract

Extracellular vesicles (EVs) play an important role in the communication between tissues and cells. However, it is difficult to screen and trace EVs secreted by specific tissues *in vivo*, which affects the functional study of EVs in certain tissues under pathophysiological conditions. In this study, a Cre-dependent CD63^flag^-EGFP co-expressed with mCherry protein system expressing mice was constructed, which can be used for the secretion, movement, and sorting of EVs from specific tissues *in vivo*. This mouse model is an ideal research tool for studying the secretion amount, target tissue, and functional molecule screening of EVs in specific tissues under different pathophysiological conditions. Moreover, it provides a new research method to clarify the mechanism of secreted EVs in the pathogenesis of the disease.

## Introduction

Extracellular vesicles (EVs) are double-layered membrane-enclosed vesicles secreted by almost all cell types and tissues. EVs have many subtypes based on their subcellular origin, size, and composition, and the most studied subtype is endosome-derived vesicles with a diameter of 50–150 nm (Lener et al., 2015; Gupta et al., 2020; Kalluri and LeBleu, 2020; Wu et al., 2020; Verweij et al., 2021).

Subsequent studies have shown that most cargos are contained in EVs, such as miRNAs, RNAs, and proteins, which mediate communication between cells and tissues through EVs delivery. They play important roles in most physiological and pathological processes such as cancer progression, metastasis, immune escape, angiogenesis (Li et al., 2013; Ridder et al., 2014; Ridder et al., 2015; Webber et al., 2015; Zhang et al., 2015; Li et al., 2016), metabolic homeostasis, inflammation, neurodegenerative diseases, and embryonic development (Zhang et al., 2010; Liu et al., 2013; Li et al., 2015; Li et al., 2020). However, the function of EVs from specific tissue sources under pathological/physiological conditions is not very clear, mainly because EVs extracted from serum are a mixture of many tissue sources. It is difficult to sort out tissue-specific EVs from the pool for further characterization and to study their function in target organs under pathological/physiological conditions.

At present, most tracing systems for EVs delivery to target tissues use intravenous injection of EVs with dye or fluorescent reporter proteins exogenously labeled *in vitro* (Ridder et al., 2014; Jaenisch et al., 2017; Thomou et al., 2017). The organotropism of exogenous EVs may be highly correlated with their secretory cell lines but cannot completely simulate the specific tissue-secreted EVs *in vivo*. Some groups have used AAV to express tissue-specific CD63-GFP fusion protein, which is an EV’s marker protein, to trace the delivery from given tissues to targeted tissues (Maguire et al., 2012; Kur et al., 2020). However, the infection efficacy of AAV also affects the judgment of the proportion of EVs from the given tissue to the total serum EVs and the evaluation of their role *in vivo* (Verweij et al., 2021).

CD63 is most widely used among syntenin or tetraspanin family members (TSPAN4, CD63, CD81, and CD9) as a reporter attached to labeling specific sEV subtypes (Verweij et al., 2021). At the same time, Bahnisikha Barman et al. found that RNA is highly enriched in certain subpopulation of small EVs which also contained CD63 (Barman et al., 2022). Men et al. used cell-type-specific ILV/exosome reporter (CD63-GFP^f/f^) mice to study the regulatory function of neuronal EVs in astrocytes. This method can effectively trace the delivery of neuronal EVs to astrocytes *in vivo*, but it still cannot solve the problem of EV screening of given tissues for characterization of their molecular components under pathophysiological conditions for functional studies (Men et al., 2019).

At present, most of the variations in the composition of EVs are characterized by exogenous EVs from 2D or 3D cell lines *in vitro* to simulate tissue secretion *in vivo* (Kim et al., 2018; Cao et al., 2019; Rocha et al., 2019; Thippabhotla et al., 2019; Verweij et al., 2019). However, cell-secreted EVs are not exactly the same as those secreted by tissues under pathological conditions *in vivo*. Newman et al. used an asialoglycoproprotein receptor1 (ASGR1) antibody to pull down liver-specific EVs, which was effective for the characterization and functional study of hepatic-secreted EVs. This approach sheds light on EV delivery between tissues. (Newman et al., 2022). However, more efforts are needed to explore and confirm the relationship between organ-specific markers and EVs. To effectively study the functions of EVs from given tissue under different pathophysiological conditions, CD63-EGFP transgenic mice were constructed in this study, and FLAG tags were inserted into the CD63 protein domain out of the EV membrane for the pull-down assay. These mice were then bred with tissue-specific Cre expression mice to obtain tissue-specific EVs tracing mice for studying communications with target tissues under different pathophysiological conditions, which can also be used to characterize specific tissue EVs to screen molecules for further functional study.

## Materials and methods

### Mice

Male C57BL/6J mice were purchased from GemParmatech (Nanjing, China). Rosa26 CAG-Loxp-stop-Loxp-CD63^Flag^EGFP-mCherry KI mice were constructed on a C57BL/6J background and bred by GemParmatech. Flag was inserted into the first transmembrane region of CD63 at nucleotide sites from 126 to 210. Villin-Cre mice were kindly gifted by Professor Jiangning Chen, Nanjing University. Alb-Cre mice were purchased from GemParmatech (Nanjing, China). Animals were group-housed as five mice per cage under a 12-h light-dark cycle with food and water provided *ad libitum.* The animals were kept in an environmentally controlled breeding room for 1 week before the experiments. All animal studies and experimental procedures were performed in accordance with the Animal Care Committee of the Nanjing University (Nanjing, China).

### Genomic PCR

Genome DNA was extracted by TIANamp Genomic DNA kit (DP304-03, Tiangen). To detected the different CD63-EGFP KI site and Cre genes locus, the PCR amplification conditions were 94°C for a 5-min initial strand separation, 40 cycles at 94°C for 45 s, 60°C for 45 s, 72°C for 1 min, and a 7-min final elongation step at 72°C. The product sizes and primers are provided at Supplementary Table S1. The amplification PCR products were separated by electrophoresis on 2% agarose gels pre-stained with GelRed (Tsingke, TSJ002). As a DNA molecular size marker, we used a DL2000 DNA marker (Tsingke, TSJ011-100).

### Histology

Tissues sections were rinsed in 4% formalin for fixation and was embedded in paraffin, and 10 μm sections were cut and stained with haematoxylin and eosin.

### Immunofluorescence

Tissue sections were blocked with 5% BSA with 0.5%Triton X-100 for 45 min, and then incubated with primary antibodies overnight. For tissues cell types analysis, anti-Villin (Santa Cruz Biotechnology, sc-58897), anti-Alblumin (abcam, ab207327), anti-SP-C (Santa Cruz Biotechnology, sc-518029), anti-nephrin (Santa Cruz Biotechnology, sc-377246) were used, and for sEV related organelles, anti-lamp2 (Santa Cruz Biotechnology, sc-18822), anti-Rab7 (Santa Cruz Biotechnology, sc-376362), anti-EEA1 (Santa Cruz Biotechnology, sc-137130) were used. After incubation with primary antibody, samples were incubated with appropriate secondary antibody (Invitrogen, Goat anti-Rabbit IgG (H + L)-Alexa Fluor Plus647, A32733 and Goat anti-Rabbit IgG (H + L)-Alexa Fluor Plus647, A32728) for 1 h and then Hoechst (Invitrogen, H1399) was used for nuclei staining.

### Confocal microscope image analysis

In the Alb-cre-CD63^Flag^EGFP-mCh-specific expression experiment, whole-body tissues were collected. Tissues were fixed with 4% paraformaldehyde for 10 h at 4°C and subsequently dehydrated in 30% sucrose for 12 h. The tissues were embedded in OCT (Servicebio, G6059) for cryostat sectioning at 8 µm. The nucleic acids in all samples were stained with Hoechst (Invitrogen, H1399). Confocal microscopy was performed using a 2-photon laser confocal microscope (Leica TCS SP8-MaiTai MP, Wetzlar, Germany).

### Exoview

Tissue specific-CD63^Flag^EGFP-mCherry mouse serum was collected from the supernatant of the blood by centrifugation at 3,000 rpm for 15 min at room temperature twice, the supernatant was then centrifuged at 12,000 rpm for 60 min. The samples were incubated overnight on microarray chips (NanoView Biosciences, Brighton, United Kingdom) coated with capture antibodies against CD9 and CD81 and negative isotype controls (hamster or rat IgG). Image and data quantification analyses were performed using the ExoView R100 platform (NanoView Biosciences).

### Serum sEV isolation

For serum sEV, serum was collected from C57BL/6J or tissue specific-CD63^Flag^EGFP-mCherryf/wt mouse supernatant of the blood by centrifugation at 3,000 rpm for 15 min at RT twice. Subsequently, the supernatant was centrifuged at × 10,000 g for 60 min to remove debris and at × 110,000 g for 2 h to obtain the sEV pellet.

### Nanoparticle tracking analysis and transmission electron microscopy

Further nanoparticle tracking analysis (NTA) of the particle concentration and size distribution of sEVs was diluted with filter cold PBS using a NanSight NS300 instrument (NanoSight Ltd.). For transmission electron microscopy (TEM) negative staining, the sEV suspension was dropped onto a copper grid with a carbon film for 3–5 min, and 2% phosphotungstic acid was dropped on a copper grid for 1–2 min until dry and then observed using an HT7800 machine (Hitachi).

### The sEV pulldown assay

Approximately, 20 ul protein A/G agarose beads (Santa Cruz Biotechnology, sc-2003) were incubated for 1 h at four°C in 500 ul PBS and 2 mg/ml BSA and were then washed with PBS three times. After washing, the beads were conjugated with anti-FLAG antibody (Invitrogen, MA1-91878) in 200 µl of PBS with continuous mixing for 4 h at four°C.The conjugated beads were then washed three times with PBS, as described above. FLAG-sEVs and unmodified sEVs obtained by differential ultracentrifugation were set to equal protein concentrations by bicinchoninic acid (BCA) quantification (Vazyme, E112-01). Beads conjugated with anti-FLAG or IgG antibody were incubated with sEVs up to a volume of 300 µl with PBS and mixed overnight at four°C. After incubation, beads were washed three times with PBS and then eluted in 30ul RIPA lysis buffer (Beyotime, P0013B) in the presence of a protease inhibitor.

### Western blots

Immunoprecipitated protein was lysed in RIPA buffer (Beyotime, P0013B) and loaded into 12% SDS-PAGE gels to be transferred onto a PVDF membrane (Millipore, IPVH00010). The primary antibody at dilutions recommended by the suppliers was anti-ASGRP1 (Santa Cruz Biotechnology, sc-52623), anti-CD63 (Santa Cruz Biotechnology, sc-5275), anti-CD9 (Santa Cruz Biotechnology, sc-13118), anti-villin (Santa Cruz Biotechnology, sc-58897), anti-GFP (Cell Signaling Technology, 2956S), anti-mCherry (Abcam, ab167453), anti-β-actin (Cell Signaling Technology, 4,970).

### Statistics

All statistical analyses were performed using the GraphPad Prism software (version 8.0; San Diego, CA, United States). Comparisons between two groups were analyzed with Student’s t-test, or more than two groups were analyzed using one-way ANOVA with the appropriate multiple comparisons test. Statistical significance was set at *p* < 0.05. Data are expressed as mean ± SEM.

## Results

### Construction of tissue specific extracellular vesicles tracing and sorting mice model

**FIGURE 1:**
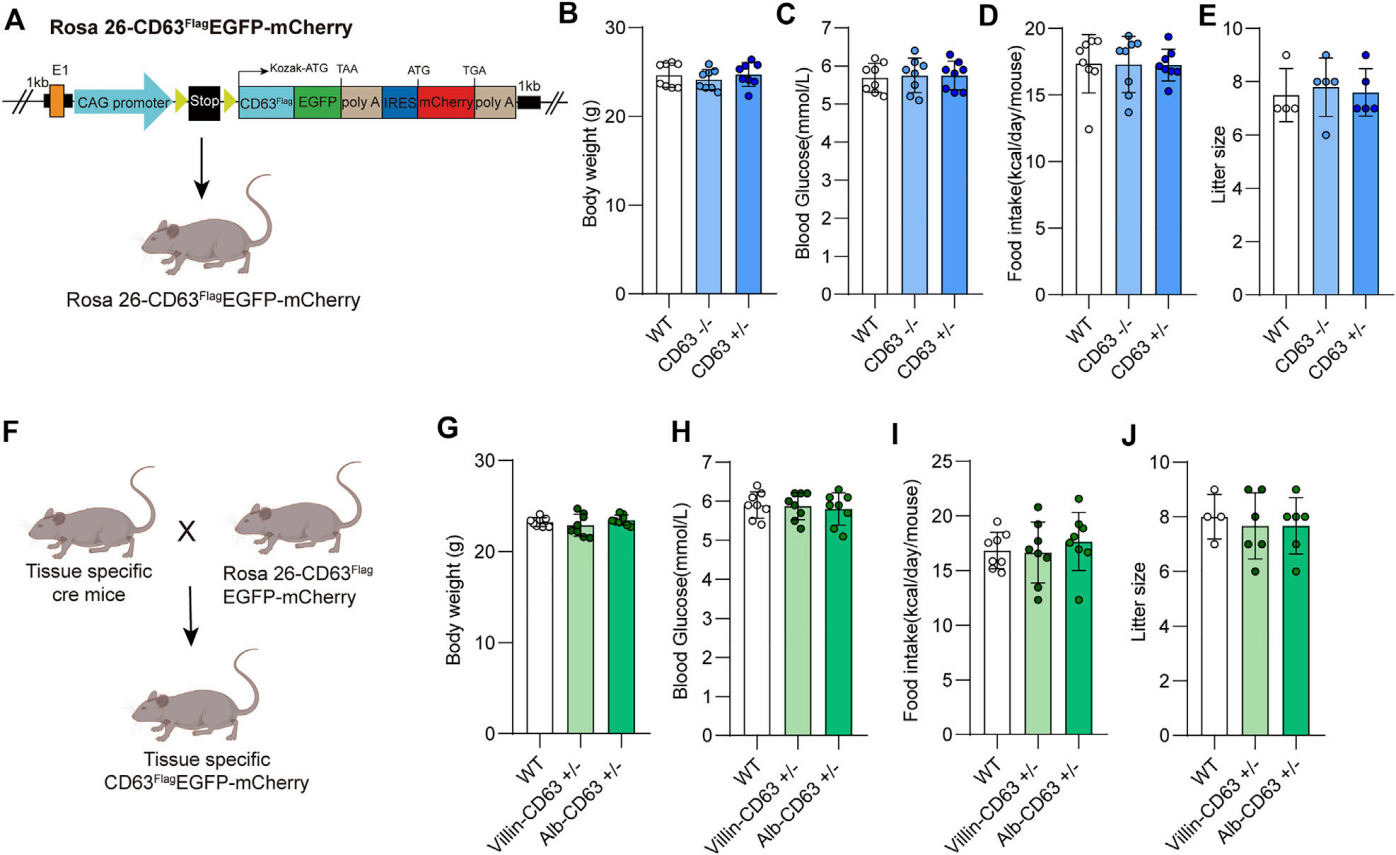
Construction and physiological assessment of tissue specific EV tracing and screening mice model. **(A)** Schematic representation of Rosa26-CD63^Flag^EGFP-mCherry KI mouse’s construction. **(B–E)** Measurements of the body weight and blood glucose, food intake, litter size from wild-type, CD63^Flag^EGFP-mCherry mouse (WT: wild-type; CD63: CD63^Flag^EGFP-mCherry) (*n* = 8 for each group). **(F)** Schematic representation of rosa26-CD63^Flag^EGFP-mCherry KI mouse crossed with tissue specific Cre mouse to generate tissue specific CD63^Flag^EGFP-mCherry mouse. **(G–J)** Physiological indicators of tissue specific CD63^Flag^EGFP-mCherry mice. Measurement of body weight, blood glucose, food intake, and litter size of wild-type and tissue-specific CD63^Flag^EGFP-mCherry mice (WT: wild-type; Alb-CD63: Alb- CD63^Flag^EGFP-mCherry; Vill-CD63: Villin-CD63^Flag^EGFP-mCherry). (*n* = 8 for each group). Data indicate the mean ± SEM.

The tetraspanin protein CD63 is a good choice as a specific marker of endosome-derived EVs because of its lower expression on the plasma membrane. We constructed a cre-dependent EV reporter expression vector by inserting a loxP-floxed stop codon upstream of the modified CD63 (extracellular domain fused with FLAG tag and the carbon terminal fused with EGFP) for EV tracing and screening, and mCherry protein was co-expressed for tracing expression tissue and efficiency. We then generated cre-dependent EV reporter (CD63^Flag^EGFP-mCherry^f/f^) mice by inserting the expression system into the Rosa26 locus of the mouse genome using Crispr-Cas9 technology (Figure 1A; Supplementary Figures S1A, B). The EV tracing system *in vivo* did not affect the physiological status of CD63^Flag^EGFP-mCherry^f/f^ mice, including litter size, body weight, blood glucose level, food intake, and litter size of both male and female offspring, compared with wild-type C57BL/6J mice (Figures 1B–E; Supplementary Figures S1C–E). After breeding CD63^Flag^EGFP-mCherry^f/f^ mice with *villin*-cre or *alb*-cre mice to obtain intestine- or liver-specific expressed EV tracing mice (Figure 1F, Supplementary Figures S2A,B), we found that the health status remained unaffected (Figures 1G–J, Supplementary Figures S2C–E). These data suggest that transgenic mice with EV tracing system are an ideal tool for tracing and screening tissue-specific EVs under pathological or physiological conditions *in vivo*.

### The tissue expressions of extracellular vesicles tracing system in the mice model

**FIGURE 2:**
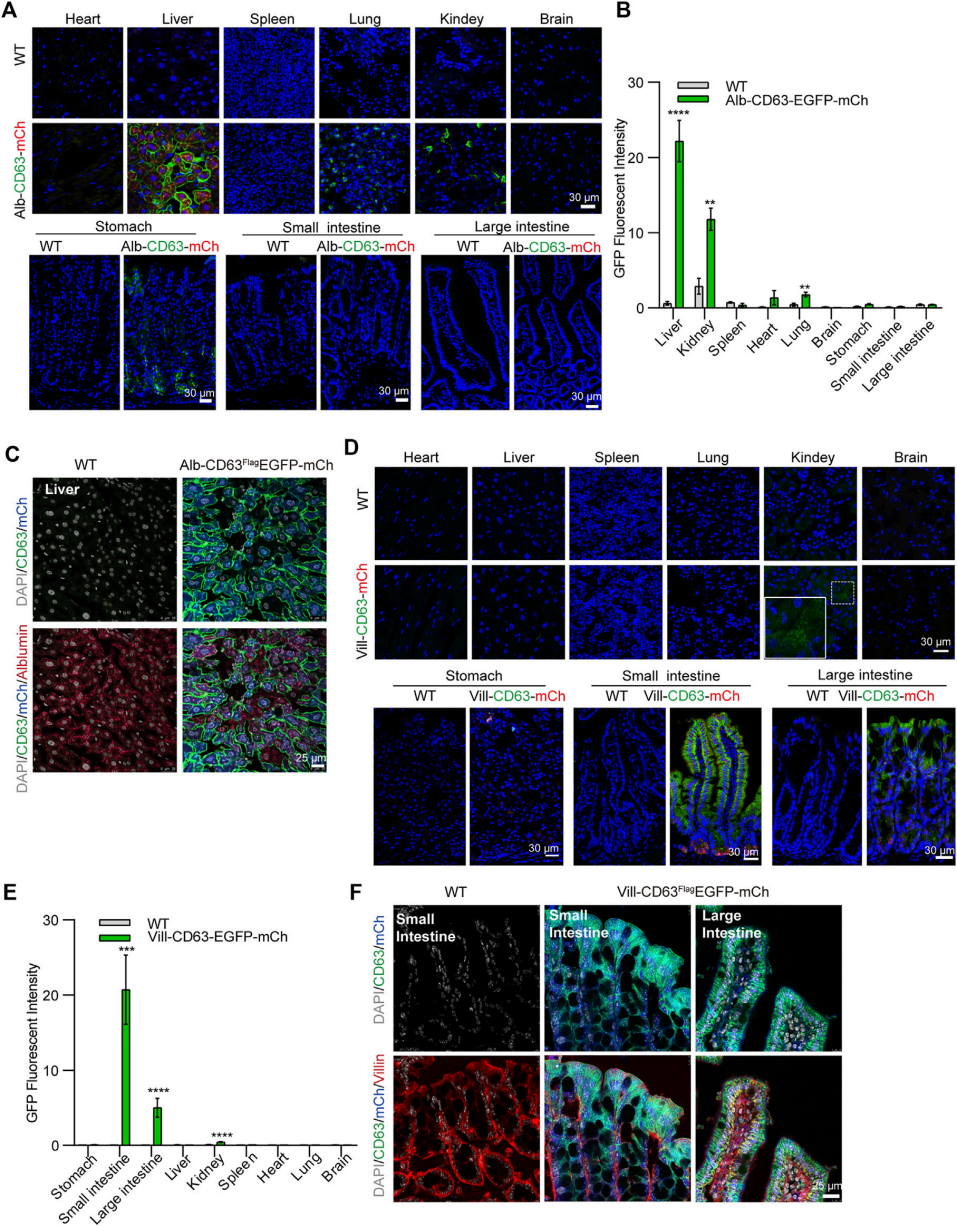
Confocal microscopic image analysis of tissue specific CD63FlagEGFP-mCherry mouse **(A)** Merged Confocal microscopy image of Alb-CD63^Flag^EGFP-mCherry mouse tissues. Blue, Nucleus; Green, GFP; Red, mCherry (Scale bar, 30 um). **(B)** Statistical analysis of GFP fluorescent intensity of each tissue of Alb-CD63^Flag^EGFP-mCherry mouse. (3 mice/group, three to four discontinuous slices for each tissue) **(C)** Immunostained cell type of liver (albumin) colocalization with EGFP and mCherry fluorescent in the liver of Alb-CD63^Flag^EGFP-mCherry mouse. Grey, Nucleus; Green, GFP; Blue, mCherry; Red, Albumin (Scale bar, 25 um). **(D)** Merged Confocal microscopy image of Villin-CD63^Flag^EGFP-mCherry mouse tissues. Blue, Nucleus; Green, GFP; Red, mCherry (Scale bar, 30 um) **(E)** Statistical analysis of GFP fluorescence intensity of each tissue of Villin -CD63^Flag^EGFP-mCherry mouse. (3 mice/group, three to four discontinuous slices for each tissue) **(F)** Immunostained cell type of intestine (villin) colocalization with EGFP and mCherry fluorescent in the intestine of Villin-CD63^Flag^EGFP-mCherry mouse. Grey, Nucleus; Green, GFP; Blue, mCherry; Red, Albumin (Scale bar, 25 um).

**FIGURE 3:**
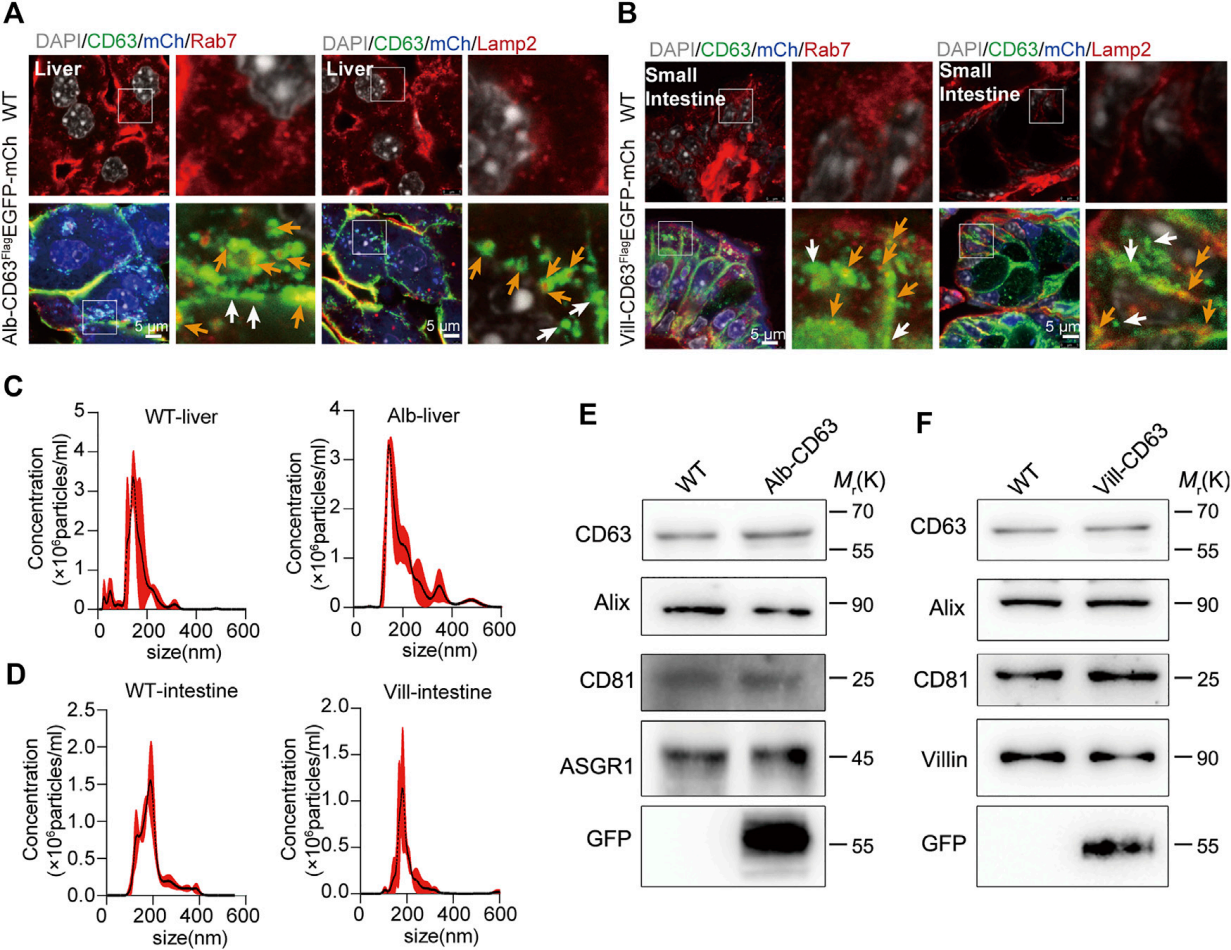
The organelles and characterization of tissue-derived EGFP EVs on tissue specific CD63FlagEGFP-mCherry mouse. **(A,B)** Immunostained organelles of MVB (Rab7) and lysosome (Lamp2) colocalization with EGFP and mCherry fluorescent in the liver of alb-CD63^Flag^EGFP-mCherry mouse **(A)** and in the intestine of Villin-CD63^Flag^EGFP-mCherry mouse **(B)**. Grey, Nucleus; Green, GFP; Blue, mCherry; Red, Rab7/Lamp2 (Scale bar, 5 um). **(C,D)** Nanoparticle tracking analysis (NTA) of EVs isolated from primary cell cultures. (WT-liver, wild-type-liver; Alb-liver, Alb-CD63^Flag^EGFP-mCherry-liver; WT-intestine, wild-type-intestine; vill-in, villin-CD63^Flag^EGFP-mCherry-intestine). (*n* = 4) **(E,F)** Western blot analysis of the protein markers of exosomes (CD63, Alix,CD9), tissue specific sEV (ASGR1, hepatic-EV specific protein; Villin, intestine-EV specific protein) and tissue specific CD63^Flag^EGFP-mCherry mouse positive sEV (GFP). Data indicate the mean ± SEM.

To determine the tissue expression of the EV reporter (CD63^Flag^EGFP-mCherry^f/f^), we detected fluorescent intensity in different tissue sections of liver-specific (alb-CD63^Flag^EGFP-mCherry^f/wt^) and intestine-specific (villin-CD63^Flag^EGFP-mCherry^f/wt^) EV tracing and screening mice, respectively (Figure 2). And all the tissues were HE-stained for further identification of EV-accumulated tissues and cells (Supplementary Figure S3). In alb-CD63^Flag^EGFP-mCherry^f/wt^ mouse tissues, we found that mCherry had an obvious expression in the liver (Figure 2A), and no expression in other tissues, suggesting that this system was specifically expressed in the liver. Additionally, the fluorescence of CD63-EGFP was significantly higher in the liver, indicating that liver EVs were labeled with CD63-EGFP (Figure 2B). However, other tissues such as the lung and kidney also showed fluorescence of CD63-EGFP without the expression of mCherry, suggesting that liver EVs may be more organotropic to these tissues under physiological conditions (Figures 2A,B). And the GFP and mCherry have a strong expression in liver cells specifically albumin (Figure 2C). Similarly, in villin-CD63^Flag^EGFP-mCherry^f/wt^ mice, except for the significant expression of mCherry and EGFP proteins in villin-expressing cell types of both the small intestine and large intestine, only the kidney showed a lower EGFP fluorescence signal (Figures 2D–F), suggesting that these tissues may be the target organs of intestinal EVs. We also conducted western blotting to detect the expression level of GFP, Flag and mCherry for further confirmation (Supplementary Figure S4A, B). The results were also consisted with the fluorescence analysis of EGFP and mCherry expression in the corresponding tissues. Through higher magnification confocal images of the CD63^Flag^EGFP-mCherry expression cells, we found that the most fluorescence of EGFP were colocalized with RAB7-positive MVB and LAMP2-positive lysosomes (yellow arrows), and a small part was isolated (white arrows), both in liver cells (from Alb-CD63^Flag^EGFP-mCherry mice, Figure 3A) and intestine cells (from villin-CD63^Flag^EGFP-mCherry mice, Figure 3B). This result also indicated that the constructed CD63^Flag^EGFP fusion protein did not affect EV generation. This EGFP labeled CD63 can be normally inserted into sEV as a tracer and screening marker. To examine the effect of CD63-EGFP/Flag-tag modification on the secretion of EVs, we compared the EV profiles including size distribution of EVs from primary cultured cells of liver and intestine from wild type and transgenic mice. The results imply that our modification of CD63 did not affect the size of EVs (Figures 3C,D). We also performed WB detection of sEV marker proteins (CD63) and tissue-specific cargo proteins (ASGR1, Villin) to further characterize the protein composition of liver or intestine specific sEVs. The hepatic-specific antibody ASGR1, as mentioned above, is an ideal indicator to identify EVs secreted from the liver, since the EVs generated from the liver have ASGR1 coated inside. And we chose villin protein for intestine specific marker. The results shown that, the marker protein was enriched in liver sEV (ASGR1) and intestine sEV (villin) (Figures 3E,F). These results were consistent with the physiological conditions of mice, suggesting that the mouse model can be used to characterize the amount of EV secreted by specific tissues under pathological or physiological conditions.

### The tracing function of tissue specific extracellular vesicles to target tissues of the mice model

**FIGURE 4:**
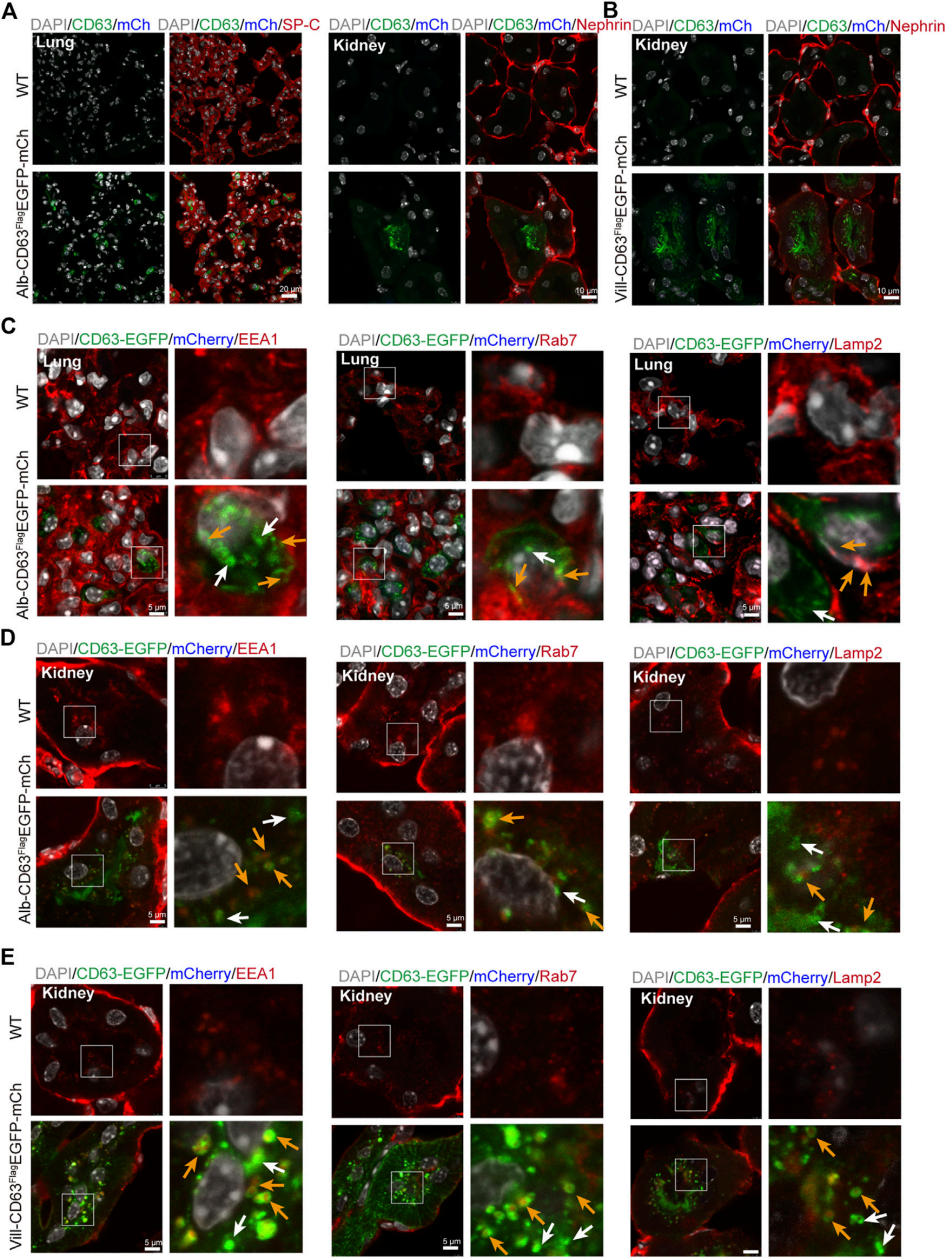
The distal organs cell type and organelles taking up EVs. **(A)** Immunostained alveolar cell (SP-C, left) and podocyte cell (Nephrin, right) colocalization with EGFP and mCherry fluorescent in the alb-CD63^Flag^EGFP-mCherry mouse. Grey, Nucleus; Green, GFP; Blue, mCherry; Red, SP-C/Nephrin. **(B)** Immunostained podocyte cell (Nephrin) colocalization with EGFP and mCherry fluorescent in the villin-CD63^Flag^EGFP-mCherry mouse. Grey, Nucleus; Green, GFP; Blue, mCherry; Red, Nephrin. **(C,D)** Immunostained organelles of lung and kidney (early endosome, EEA1; MVB, Rab7; lysosome, Lamp2) colocalization with EGFP and mCherry fluorescent in the alb-CD63^Flag^EGFP-mCherry mouse. Grey, Nucleus; Green, GFP; Blue, mCherry; Red, Rab7/Lamp2/EEA1 (Scales bar, 5 um). **(E)** Immunostained organelles of kidney (MVB, Rab7; lysosome, Lamp2; early endosome, EEA1) colocalization with EGFP and mCherry fluorescent in the villin-CD63^Flag^EGFP-mCherry mouse. Grey, Nucleus; Green, GFP; Blue, mCherry; Red, Rab7/Lamp2/EEA1 (Scales bar, 5 um).

To verify whether this mouse model can be used to track specific tissue EVs’ delivery to target tissues *in vivo*, we selected lung and kidney tissues with stronger EGFP fluorescence signal in alb-CD63^Flag^EGFP-mCherry^f/wt^ mice, and kidney tissues in villin-CD63^Flag^EGFP-mCherry^f/wt^ mice for further study. To make clear the distant tissues cell types which take up those sEVs, we used IF detection to show that liver sEVs were mostly taken up by alveolar specific-cells (pulmonary surfactant protein C, SP-C expressed) in lung (Glasser et al., 2005), and podocyte-related cells (Nephrin expressed) in kidney (Kostovska et al., 2022) of the liver specific sEV tracing mice (Figure 4A). In lung tissue, there are still a few other cells types that do not express SP-C also show EGFP fluorescence, and these cell types still need to be further verified. Similarly, as a target tissue for intestine-specific EVs, we found that podocyte-related cells in the kidney remained the dominant cell type in taking up of intestine sEVs, as shown in Figure 4B. In the target tissues, we also found EGFP-labeled EVs were colocalized with EEA1-positive early endosomes, RAB7-positive MVBs and LAMP2-positive lysosomes (yellow arrows), and there were still many sEV isolated (white arrows) in lung and kidney of the Alb-CD63^Flag^EGFP-mCherry mice (Figures 4C,D). The same pattern was seen in the kidney (Figure 4E), the target tissues of villin-CD63Flag^EGFP^-mCherry mice. Interestingly, we also found that many EVs were still intact vesicles in the target tissue cells, particularly in the kidney cells.

### Proportion analysis function of tissue specific extracellular vesicles in serum of these mice model

**FIGURE 5:**
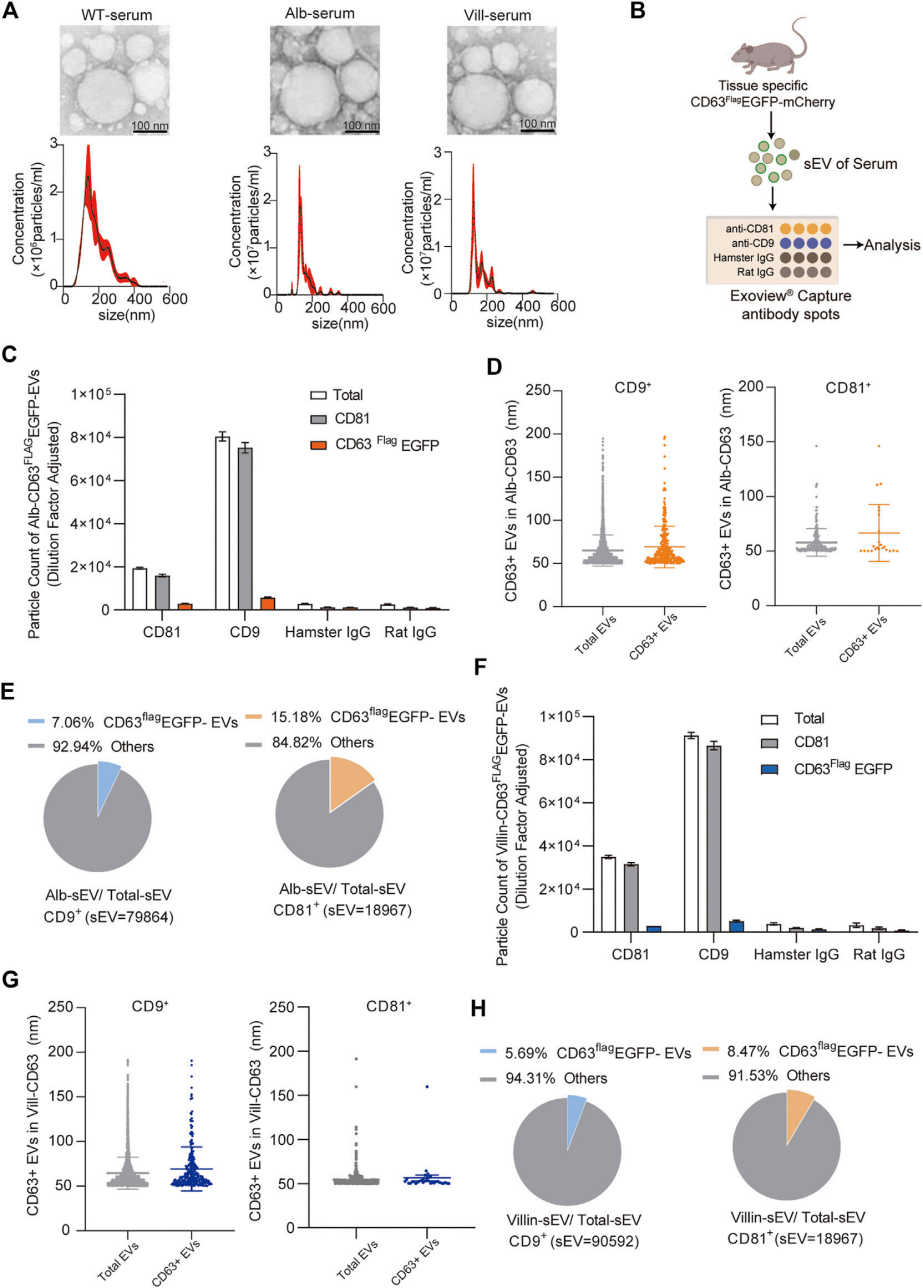
Characterization and quantification of tissue-specific CD63^Flag^EGFP-mCherry serum EV **(A)** Nanoparticle tracking analysis (NTA) and transmission electron microscopy (TEM) of EVs isolated from mouse serum (WT, wild-type; Alb, Alb-CD63^Flag^EGFP-mCherry; vill, villin-CD63^Flag^EGFP-mCherry). (*n* = 4) **(B)** Schematic representation of *ExoView* microarray analysis of the serum of tissue specific-CD63^Flag^EGFP-mCherry. (*n* = 3, collected serum sEV mixed to one sample for Exoview detection) **(C)** Particle counts of serum sEVs captured by CD81 and CD9 antibodies respectively using ExoView technology from Alb-CD63^Flag^EGFP-mCherry mouse. **(D)** The size of CD63^Flag^-EGFP^+^ sEVs captured by CD9 antibody (left)and CD81 antibody (right)using Exoview technology from Alb-CD63^Flag^EGFP-mCherry mouse serum. **(E)** Pie charts representing percentages of Alb-sEVs in total sEVs from CD9 antibody-captured (left) and CD81 antibody-captured (right) serum-sEVs. **(F)** Particle counts of serum sEVs captured by CD81 and CD9 antibodies respectively using ExoView technology from Villin-CD63^Flag^EGFP-mCherry mouse. **(G)** The size of CD63^Flag^-EGFP^+^ sEVs captured by CD9 antibody (left)and CD81 antibody (right)using Exoview technology from villin-CD63^Flag^EGFP-mCherry mouse serum. **(H)** Pie charts representing percentages of Villin-sEVs in total sEVs from CD9 antibody-captured (left) and CD81 antibody-captured (right) serum-sEVs.Data indicate the mean ± SEM.

To further confirm whether tissue-specific CD63^flag^-EGFP^+^ EVs in this mouse model can represent the content of EVs secreted by the given tissue *in vivo*, we first used NTA and TEM to analyze the size of EVs in serum, and the results showed that there was no significant change in the size range of total EVs from serum (Figure 5A). The peak value was around 150nm, and there were also many large vesicle peaks, which was correlated with the diversity of vesicle types in serum. Then, we analyzed the proportion of CD63^flag^-EGFP^+^ EVs in the serum of alb-CD63^Flag^EGFP-mCherry^f/wt^ and villin-CD63^Flag^EGFP-mCherry^f/wt^ mice using ExoView analysis (Figure 5B). ExoView technology can use the mouse EV marker proteins CD9 and CD81 antibodies to sort out CD9^+^ or CD81^+^ EVs in serum and count the number of exosomes containing the fluorescence signal of CD63^flag^-EGFP^+^ EVs (Harkonen et al., 2019; Khan et al., 2021). Using this technique, we found that the size of the CD63^flag^-EGFP^+^ EVs did not change in both CD9^+^ and CD81^+^ EVs in both alb-CD63^Flag^EGFP-mCherry^f/wt^ mice and villin-CD63^Flag^EGFP-mCherry^f/wt^ mice (Figures 5C,D,F,G). And the proportion of CD63^flag^-EGFP^+^ EVs in total CD9^+^ or CD81^+^ EVs was 7–15% respectively in alb-CD63^Flag^EGFP-mCherry^f/wt^ mice (Figure 5E), which is close to the proportion of liver EVs reported before pull-down with liver-specific ASGR1 antibody from serum total EVs, suggesting that this technique can be effectively used for analyzing tissue-specific secreted EVs (Newman et al., 2022). Similarly, using this technique, we found that intestinal EVs accounted for 5.7% and 8.4% of serum CD9^+^ and CD81^+^ EVs in villin-CD63^Flag^EGFP-mCherry^f/wt^ mice, respectively (Figure 5H). However, what is more puzzling is that the peak size of EVs secreted by primary hepatocytes and intestinal cells detected by NTA was around 150 nm (Figures 3C,D), which was different from the size of EVs detected by ExoView (Figures 5D,G). This may be due to the sensitivity of different detection methods.

### Sorting function of tissue specific extracellular vesicles from total serum extracellular vesicles of these mice model

**FIGURE 6:**
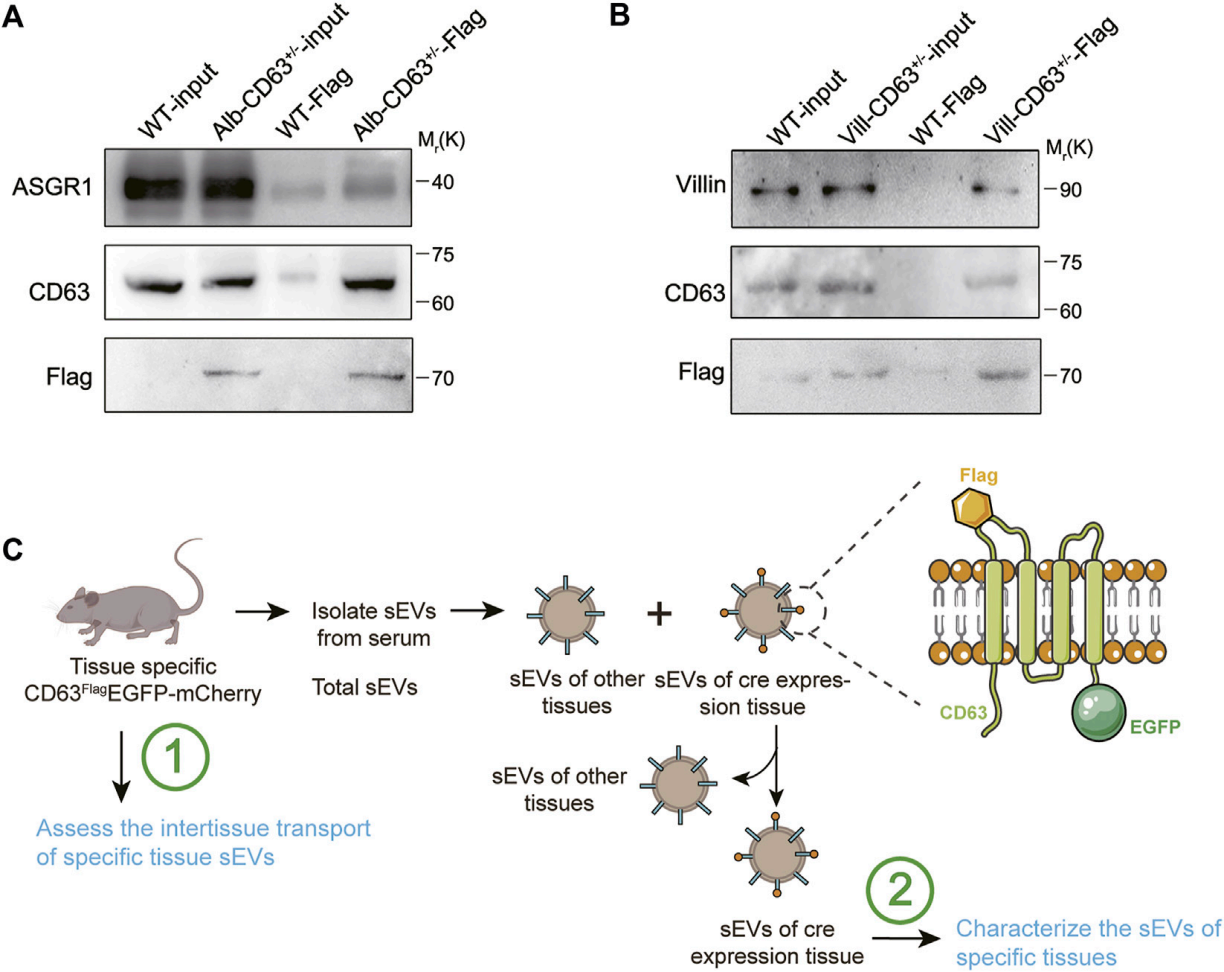
Western blot analysis of serum sEVs immunoprecipitated with anti-Flag antibody. **(A)** Representative western blot results of ASGR1 (hepatic-EV specific protein), CD63 and Flag *via* flag-specific immunoprecipitation from both wild-type and alb-CD63^Flag^EGFP-mCherry mouse serum EVs. **(B)** Representative western blot results of villin (intestine specific protein), CD63 and Flag *via* flag-specific immunoprecipitation from both wild-type and villin-CD63^Flag^EGFP-mCherry mouse serum EVs. **(C)** Schematic representation of sEVs from tissue specific CD63^Flag^EGFP-mCherry mouse isolated for testing.

To further explore whether these mouse models can be used for screening of tissue-specific secreted EVs, we took alb-CD63^Flag^EGFP-mCherry^f/wt^ mice for further study and used flag antibody-labeled agarose beads to separate hepatic EVs from total EVs extracted from serum. Compared with the equal amount of input groups, a stronger band of ASGR1 was observed in the alb-CD63^Flag^EGFP-mCherry^f/wt^ mice EVs pulled down with FLAG antibody, suggesting that antibody-enriched EVs may be secreted from the liver (Figure 6A). We also detected the enrichment of villin protein in the villin-CD63^Flag^EGFP-mCherry^f/wt^ mice EVs obtained by the same method (Figure 6B). These results suggest that our mouse models may provide a new method for screening tissue-specific EVs for further characterization and screening of their active components. These results suggest that mouse models may provide a new method for screening tissue-specific EVs for further characterization and screening of their active components.

## Discussion

As an important component of intercellular communication between tissues, EV participate in the regulation of almost all physiological and pathological processes. However, owing to the limitations of current research conditions, it is difficult to screen EVs secreted by specific tissues to study their molecular components, such as proteins, RNAs, miRNAs, and other molecules, and how EVs target organ selectivity, which may be crucial to the function of these EVs under pathophysiological conditions. The tissue-specific EV tracing and screening mouse model constructed in this study could effectively solve this problem (Figure 6C).

First, these mouse models can effectively represent the *in vivo* conditions of EVs secreted by tissues. Most previous studies used AAV to express CD63-GFP to represent the EVs of specific tissues, but this technology can only trace the movement of the given tissue EVs *in vivo* (Takahashi et al., 2013; Lai et al., 2014; Zomer et al., 2015). Therefore, it cannot represent the real quantity of EVs in the whole tissue, owing to the uneven expression of AAV. The mice constructed in this study used tissue-specific Cre expression mice bred with CD63 mice to obtain a uniformly expressed CD63^Flag^-EGFP-mCherry system in specific tissues. Moreover, CD63 gene editing did not affect the size, secretion of the EVs and uptake of the target tissues. The EVs proportions of liver-secreted EVs calculated by ExoView technology were very similar to those of EVs pulled down by the liver-specific expression protein ASGR1, indicating that tissue-specific EVs labeled in these mouse models can effectively simulate the condition of given tissue-secreted EVs *in vivo*.

Second, this mouse model visualizes the secretion and movement of EVs from specific tissues *in vivo*. Most of the current studies used CD63-EGFP fusion protein to track EV delivery *in vivo*, but the occurrence of the fluorescence signal of CD63-EGFP in other tissues raises the question of whether this disturbance was caused by the delivery of EVs secreted by specific tissues to the recipient tissues or the non-specific expression of the tracing system. In this study, the EV tracing system and mCherry protein were co-expressed, which could effectively solve this problem. Tissue cells with a fluorescence signal of CD63-EGFP can be determined as target tissues delivered by EV if mCherry is not expressed. For example, in this study, intestinal EVs had an obvious affinity for Kidneys, while liver EVs had an obvious affinity for lungs and kidneys. At the same time, we also found intact EVs in the target tissue cells, especially renal tissue cells, by high-resolution confocal images. These results suggest that some EVs may be taken up by endocytosis, rather than releasing their cargoes directly by membrane fusion. In addition, in these mouse models, we also found that the intestinal crypt cells had a higher EVs secretory capacity because of the higher expression of mCherry and lower CD63-EGFP levels in the crypt cells than in the intestinal epithelial cells. This result is consistent with the strong EV-secreting ability of intestinal stem cells reported previously. However, this model cannot be used to trace the delivery of EVs to the given tissue for expression of CD63-EGFP itself.

Finally, these mouse models were able to identify EVs secreted by specific tissues. Flag antibody-coupled beads could easily pull-down specific tissue-secreted EVs with flag tags fused to the outer membrane domain of CD63. This EV screening model can be applied to most tissue-specific EVs screening by breeding with tissue-specific Cre expression mice, eliminating the complicated process of screening certain tissue EV marker proteins in the methods of screening certain tissue EVs with antibody pulled down of the EV membrane proteins secreted by specific tissues. The molecular composition of EVs screened by this method can be further analyzed to study the compositional variations of the given tissue EVs under different pathophysiological conditions, providing a basis for studying the communication and regulation functions with other tissues under different pathological or physiological conditions.

Therefore, in this study, we provide an ideal tool for studying the secretion, delivery, and compositional characterization of specific tissue-secreted EVs under different pathophysiological models, which provides a new method for studying the molecular mechanism of specific tissue-secreted EVs during the pathogenesis of various diseases.

## Data Availability

The original contributions presented in the study are included in the article/Supplementary material, further inquiries can be directed to the corresponding authors.
