# Supplementary material for: Construction of a mouse model that can be used for tissue-specific EV screening and tracing in vivo
Source: Front Cell Dev Biol. 2022 Nov 18;10:1015841. doi: 10.3389/fcell.2022.1015841 (PMC9715976; doi:10.3389/fcell.2022.1015841)
Supplement: Supplementary file 1 [file DataSheet1.docx]

**Supplementary data**

**Construction of a mouse model that can be used for tissue-specific EV screening and tracing *in vivo***

Weili Li^1,2†^, Jin Wang^1,2†^, Xiaojiao Yin^1†^, Huanhuan Shi^1,2†^, Benben Sun^1^, Mengru Ji^1,2^, Huichen Song^1，2^, Jiachen Liu^1^, , Yihao Dou^1^, Chenghong Xu^1^, Xiaohong Jiang^1,2^, Jing Li^1,2^, Liang Li^*1,2^, Chen-Yu Zhang^*1,2^, Yujing Zhang^*1,2^

**File list:**

Figure S1

Figure S2

Figure S3

Figure S4

Table S1

**Supplementary Figure**

**Fig. S1**

**
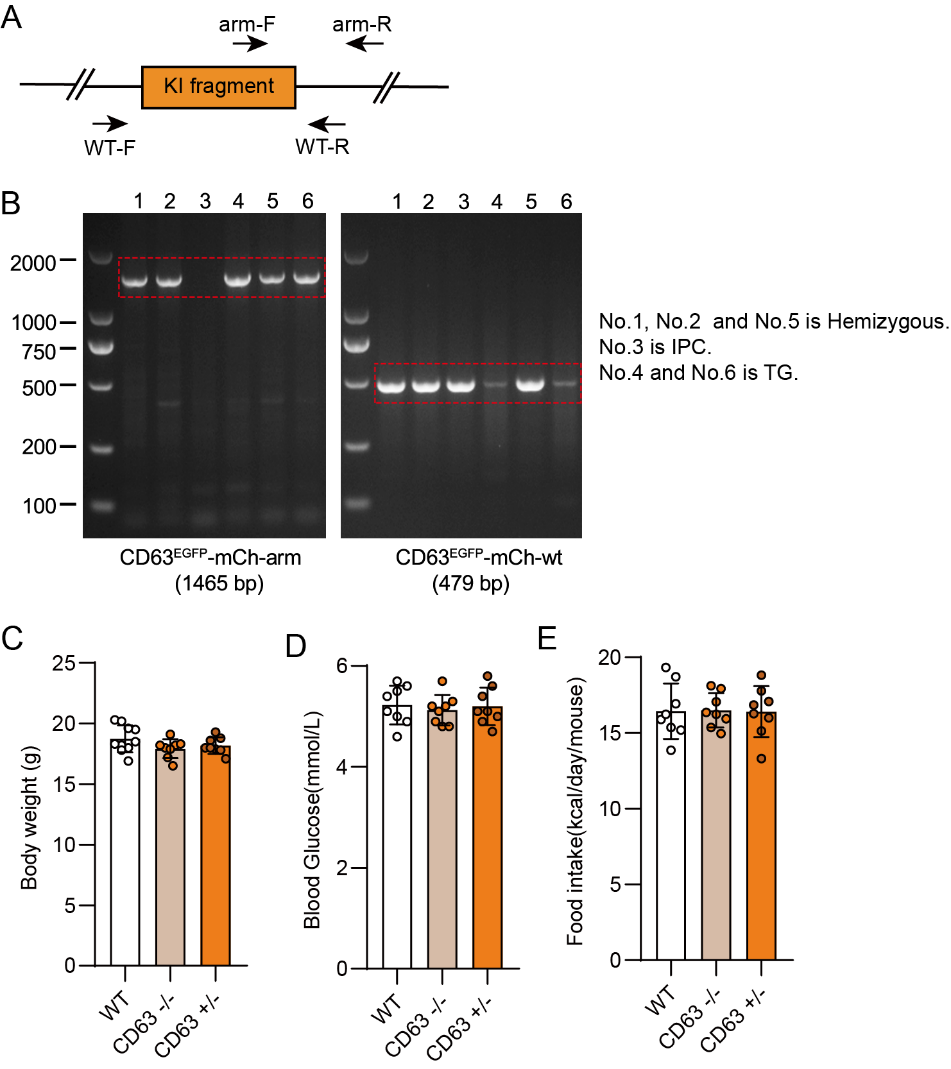
**

**Supplementary figure 1** Genotyping of CD63^Flag^EGFP-mCherry mouse.

**A** Genotyping scheme of the KI fragment, primers designed for both the knock-in arm and wild-type sequence along with their locations are displayed. (F: forward primer; R: reverse primer.)

**B** Genotyping results of the constructed mice via agarose gel electrophoresis, PCR product of strands with knock-in arm and strands with wild-type sequence will be 1465bp and 479bp, respectively. (n=6)

**C-E** Measurements of the body weight, blood glucose and food intake of wild-type and CD63^Flag^EGFP-mCherry female mouse (WT, wild-type; CD63, CD63^Flag^EGFP-mCherry). (n=6 to 8 for each group).

Data indicate the mean ± SEM.

**Fig. S2**


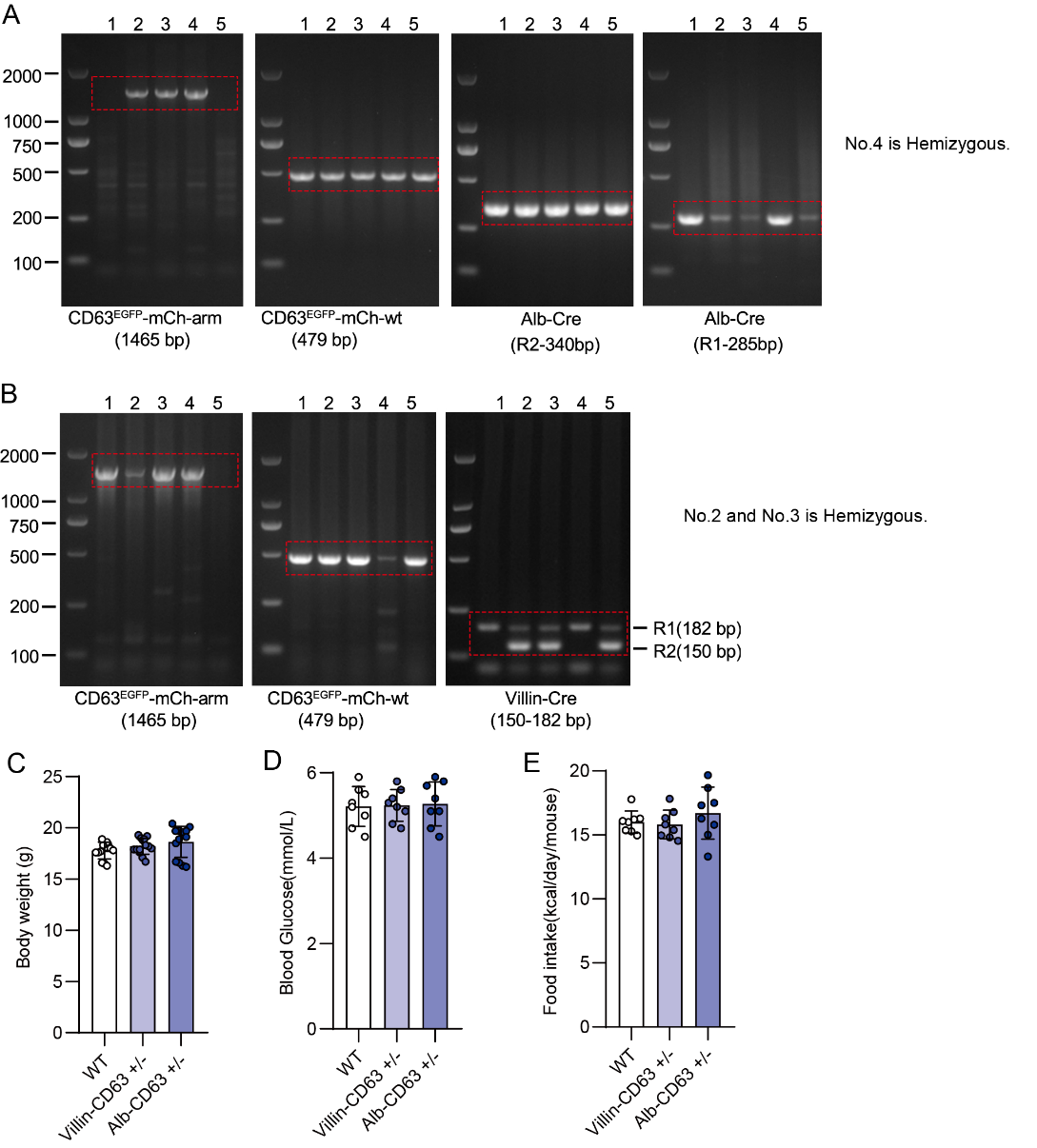


**Supplementary figure 2** **Genotyping of tissue specific CD63^Flag^EGFP-mCherry mouse.**

**A-B** Genotyping results of tissue specific CD63^Flag^EGFP-mCherry mouse via agarose gel electrophoresis, PCR product of strands with Alb-CD63 (**A**) and Vill-CD63 (**B**) Cre mouse and CD63^Flag^EGFP-mCherry respectively.

**C-E** Measurements of the body weight, blood glucose level, food intake of WT and tissue specific CD63^Flag^EGFP-mCherry female mouse (WT, wild-type; Alb-CD63, Alb-CD63FlagEGFP-mCherry; Vill-CD63, Villin-CD63FlagEGFP-mCherry)**.** (n=6 to 8 for each group). Data indicate the mean ± SEM.

**Fig. S3**

**
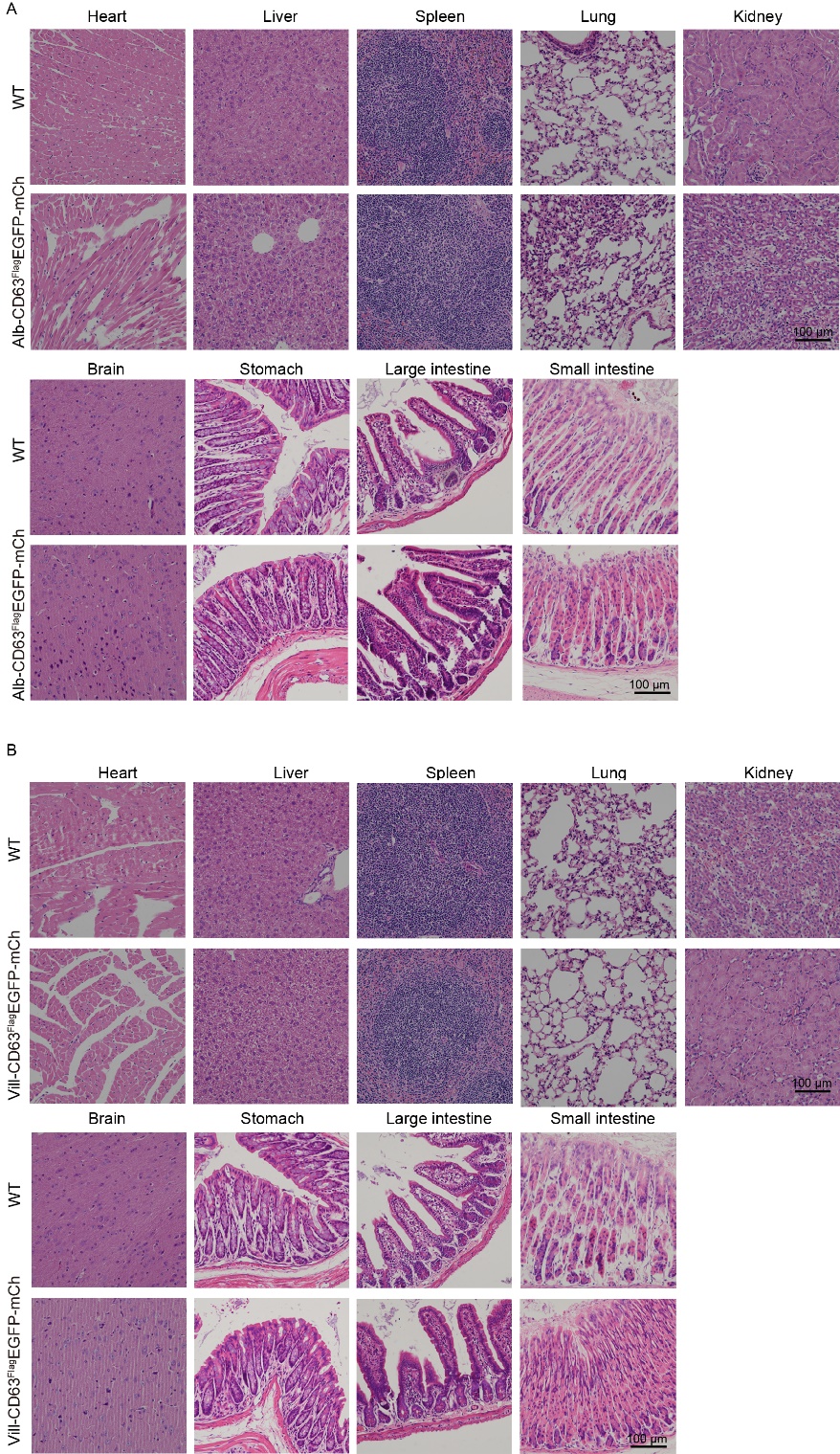
**

**Supplementary figure 3 H&E staining imaging analysis of tissue specific CD63FlagEGFP-mCherry mouse.** Representative H&E staining microscopy image of Alb-CD63^Flag^EGFP-mCherry (A) and Villin-CD63^Flag^EGFP-mCherry (B) mouse in different organs, including liver, kidney, spleen, heart, lung, brain, stomach, small intestine and large intestine.

**Fig. S4**

**
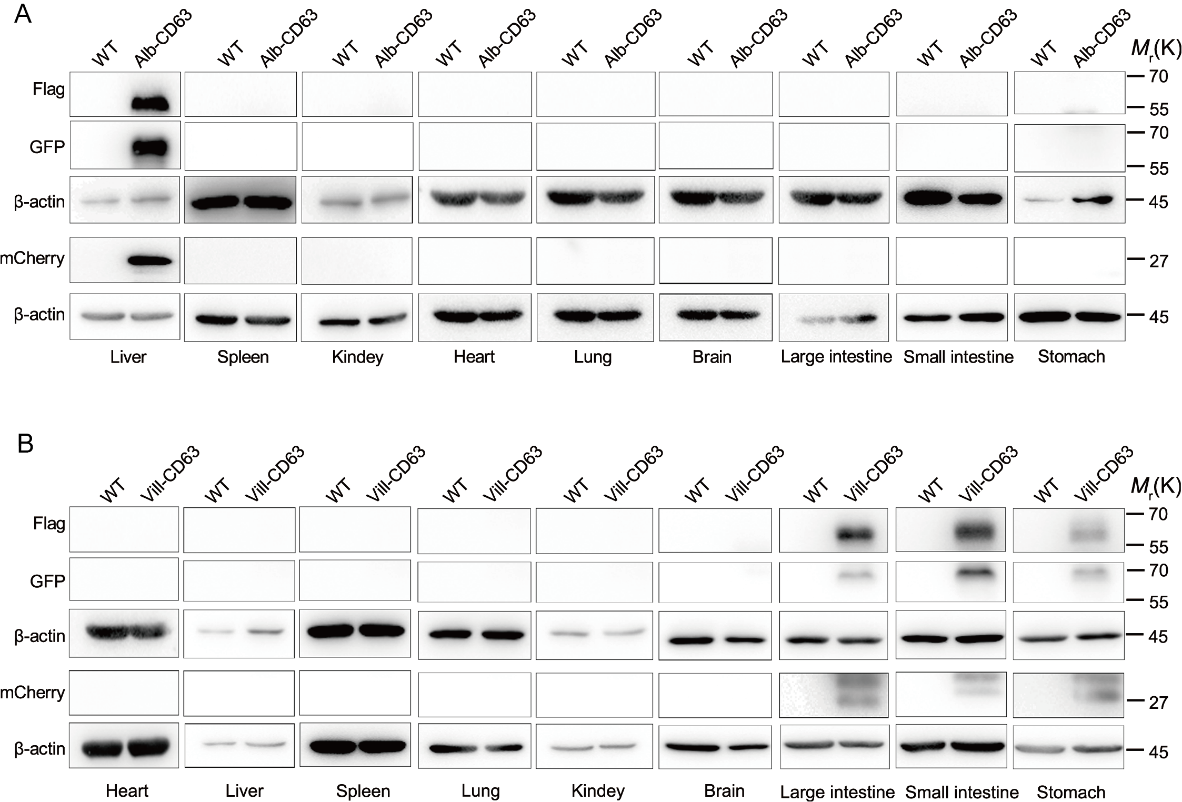
**

**Supplementary figure 4** Genotyping results of the tissue specific CD63^Flag^EGFP-mCherry mouse via western blot.

Representative western blot results of Flag, GFP, β-actin and mCherry in different organs (including liver, kidney, spleen, heart, lung, brain, stomach, small intestine and large intestine) of both wild-type, Alb-CD63FlagEGFP-mCherry (**A**) and Villin-CD63FlagEGFP-mCherry (**B**). Independent trials of both Flag, GFP and β-actin as well as mCherry and β-actin were carried out respectively.

**Supplementary Table1** Genotyping primer for transgenic mouse.

|  | Forward primers | Reverse primers | Band size |
| --- | --- | --- | --- |
| Alb-Cre | GGGCAGTCTGGTACTTCCAAGCT | R1:TAGCTACCTATGCGATCCAAACAAC  R2: ATATCCCCTTGTTCCCTTTCTGC | 340 bp  285 bp |
| Villin-Cre | GCCTTCTCCTCTAGGCTCGT | R1: TATAGGGCAGAGCTGGAGGA  R2: AGGCAAATTTTGGTGTACGG | 182 bp  150 bp |
| CD63^EGFP^-mCh-arm | TGGCGTTACTATGGGAACATACGTC | CTTGAACTCCGAGCCACCTCTC | 1465 bp |
| CD63^EGFP^-mCh-wt | CCCAAAGTCGCTCTGAGTTGTTA | TCGGGTGAGCATGTCTTTAATCT | 479 bp |

Alb-Cre: TG= ~340 bp; Hemizygous = ~285 bp and 340 bp; IPC = 285 bp.

Villin-Cre: TG= ~150 bp; Hemizygous = ~150 bp and 182 bp; IPC = 182 bp.

CD63^EGFP^-mCh: TG= ~1465 bp; Hemizygous = ~479bp and1465 bp; IPC = 479 bp.
